# Perceived Stress, Stressors, and Coping Mechanisms among Doctor of Pharmacy Students

**DOI:** 10.3390/pharmacy3040344

**Published:** 2015-11-25

**Authors:** Jennifer W. Beall, Renee M. DeHart, Robert M. Riggs, John Hensley

**Affiliations:** McWhorter School of Pharmacy, Samford University, 800 Lakeshore Drive, Birmingham, AL 35229, USA; E-Mails: jwbeall@samford.edu (J.W.B.); rmriggs@samford.edu (R.M.R.); jhensley@samford.edu (J.H.)

**Keywords:** perceived stress, stressors, coping mechanisms, student pharmacists

## Abstract

The primary purpose of this study was to examine perceived stress in doctor of pharmacy students during their first, second, and third years of their program in a fully implemented new curriculum. The secondary objectives were to determine if there is a relationship between perceived stress and certain demographic variables, to compare student pharmacist perceived stress to the perceived stress in the general population, and to examine student reported stressors during pharmacy school and coping strategies employed for those stressors. A previously validated survey (Perceived Stress Scale-10) was given to first, second, and third year student pharmacists. Females exhibited higher mean stress scores than males. The under 22 years and over 32 years age categories exhibited higher mean stress scores than the 22 to 26 year old student population. There was no significant difference in perceived stress scores between classes of the program. Only a portion of the variation in stress scores was predicted by gender, age, marital status, race, and year in curriculum. Stress scores among these student pharmacists are higher overall than those in previously published probability samples in the general population. Class assignments and completing electronic portfolios were the top stressors reported. Spending time with family and friends was the most frequent coping mechanism reported. Programming related to stress reduction (particularly among female and nontraditional age students) appears warranted.

## 1. Introduction

Stress can be thought of as a state resulting from an “imbalance between demands and resources” or as occurring when “pressure exceeds one’s perceived ability to cope” [1]. While stress is often associated with negative events or problems in a person’s life, it can also result from positive life occurrences, such as enrolling in professional programs to start/advance one’s career. Stress in college students can be triggered from a variety of issues including relationships, academics, and financial concerns [2]. Stress in student pharmacists is significant, because a correlation between increased stress and decreased health-related quality of life has been observed in at least one study of student pharmacists [2].

Accreditation Council for Pharmacy Education (ACPE) accreditation standards and guidelines specifically contain guidance for Standard 24.e regarding student stress: “Colleges and schools are encouraged to assess and correct underlying causes of ineffective learning experiences. Such assessments consider the amount of student effort, the quality of faculty teaching, and the appropriateness of learning assessments used within the courses. In this regard, these assessments include measurements of perceived stress in faculty, staff, and students and an evaluation of stress’ potential for a negative impact on programmatic outcomes and morale.” [3]. While these accreditation standards have been updated for 2016, student affairs’ professionals still work regularly to improve/enhance student wellness during pharmacy school. Management of non-academic issues like stress is important for academic success [4].

Despite ACPE recommendation and several tools being available, little literature is actually published examining stress levels in student pharmacists [2,4,5,6,7,8,9,10,11]. Two studies that have examined student pharmacist stress verses other health professional students stress yielded conflicting results [7,9]. Henning *et al.*’s research demonstrated student pharmacists’ stress level on average was higher than medical students [7], while Abhay’s *et al.*’s research demonstrated the opposite [9]. There is also conflicting data on whether female student pharmacists experience more stress than their male counterparts [2,4] and whether certain years of the pharmacy school experience are more stressful than others [4,10].

A variety of assessment tools are available and have been used to examine stress in student pharmacists [2,4,5,6,7,8,9,10,11,12,13,14,15]. These tools include the Perceived Stress Scale (PSS), the Student-Life Stress Inventory, the Beck Stress Inventory, the Derogatis Stress Profile, and a survey adapted from a nurse practitioner tool [2,4,5,6,7,8]. The most commonly used tool for assessing student pharmacist stress to date is the Perceived Stress Scale (PSS). The PSS is available as a 10 (PSS-10) or 14 (PSS-14) question tool [12]. At least five investigator groups have used the PSS to assess stress among student pharmacists [2,9,10,11,14]. The one study to date using the PSS-10 revealed that women reported higher stress than men and that year in program correlated negatively with stress levels. This study yielded a mean PSS-10 score in female students of 19.0 and of 17.26 in male students [14]. These prior studies (particularly those of Cohen and Votta) provide initial data for other programs to benchmark to moving forward as they assess student stress. This current study will be the second study published to date that uses the PSS-10 in U.S. student pharmacists. Based on previous studies (gender) and faculty perceptions (age of student), subgroup comparisons were made in this study to examine differences in student stress based on gender and age. Since the school’s curriculum was changed in 2009 to include the use of horizontal integration of course content (integration of course content within the same year) and the use of e-portfolios, examining these areas, and their contribution to student stress were also of particular interest to the investigators. Information from this study will also help provide a benchmark for other pharmacy programs’ student affairs professionals who may be evaluating which stress assessment tool to use in practice.

The primary purpose of this study was to examine perceived stress in student pharmacists during their first, second, and third years of the doctor of pharmacy program in a fully implemented new curriculum. In 2009, the school implemented an integrated curriculum model that included intentional sequencing of content in the pharmaceutical sciences and pharmacotherapy courses, weekly integrated pharmacy application laboratories, introductory pharmacy practice experiences (IPPEs) starting in week five of the first professional year, and electronic portfolios. We wanted to explore which, if any of these, significantly contributed to stress among students. We also wanted to determine if intentional sequencing of course content across multiple courses in our new curriculum led to differing or similar levels of perceived stress across years in the didactic curriculum. Additional secondary objectives of this study were to determine if there is relationship between perceived stress and certain demographic variables (gender, year in curriculum, and age), to compare student pharmacist perceived stress to the perceived stress in the general population (as reported in previously published literature), and to examine student reported triggers for stress during pharmacy school and coping strategies employed for those stressors.

## 2. Methods

### 2.1. Materials

This study was approved by Samford University’s Institutional Review Board. First, second, and third year student pharmacists at one private university were asked to complete a survey during a class meeting during the spring semester 2012. The survey included demographic information and the 10 item PSS. The PSS is available as a 10 (PSS-10) or 14 (PSS-14) question tool [12]. The PSS-10 is a validated stress instrument and does not lose any psychometric quality when compared with the longer version but is shorter and takes less time to complete [13]. Additionally, the PSS-10 has been used in large probability samples, allowing for the comparison of pharmacy students to a more general, non-pharmacy based population [13].

### 2.2. Procedures

Participation was voluntary and anonymous. Participants received information about the survey at the time of survey completion in accordance with usual Institutional Review Board (IRB) expectations at the institution. Participants were not compensated or incentivized in any way for their participation. The paper survey included the 10 item PSS, demographic questions, and questions concerning stressors and relievers (derived from the study by Marshall *et al.*) [2]. Based on previous studies showing higher stress scores in female pharmacy students [2], it was decided prospective to compare female and male stress scores to each other. Likewise, the study was prospectively designed to examine stress scores based on age (due to faculty perceptions of higher stress among younger students at the institution). For stress, trigger and alleviator questions responses of “very often”, “fairly often” or “sometimes” were considered a “yes” response to the question.

### 2.3. Data Analyses

All data were entered and analyzed in Microsoft Excel 2010. Chi square tests were used when comparing nominal data. *T*-tests were used for comparisons of continuous data between two groups (gender) and ANOVA tests were used when comparing three or more groups (year in the curriculum, age groups).

## 3. Results

Two hundred and forty two students completed the survey (response rate 68.4%). Demographics can be seen in Table 1. The composition of the sample is very similar to the entire student body in regards to gender, age, race, and marital status. PSS score by class, age, and year in curriculum can be seen in Table 2. Due to similarities in the stress score results of the age 22 to 26 and 27–31 groups, as well as similarities in the stress score results for the age 31–36 and 37 and over groups, these groups are combined in our results section. Further examination of the age range categories revealed a bimodal distribution in terms of mean PSS scores. The under 22 years of age and over age 32 categories exhibited higher mean perceived stress scores than those students aged 22–32 (*p* = 0.01). While *t*-test results showed female students reporting higher levels of perceived stress (Table 3) compared to their male counterpart (*p* = 0.0001), there was no statistical difference in perceived stress between Caucasian, African American, Hispanic, Asian, and Native American students. There was no statistical difference in perceived stress between the P1, P2 and P3 classes (Table 3; *p* = 0.123). The perceived stress scale (PSS) questionnaire results of the overall group can be seen in Table 2.

Multiple linear regression including include age, race, pharmacy year, marital status, dependent status, previous education level, and gender (as independent variables) was conducted to explore associations with stress level. Using these variables in the regression model yielded an R-squared of 0.13, indicating these variables only predict a small amount of the variation of stress scores among students.

**Table 1:** Demographics for survey participants (*n* = 242).

Characteristic	No. (%)
**Sex**	
Male	72 (29.8)
Female	170 (70.2)
**Marital Status**	
Single and not in a committed relationship	90 (37.2)
In a committed relationship	94 (38.8)
Married	58 (24.0)
Divorced or separated	0 (0)
**Have Children ^a^**	
Yes	23 (9.6)
No	217 (90.4)
**Highest completed Education Level ^b^**	
No degree	69 (28.6)
Bachelor’s degree	64 (26.6)
Master’s degree	102 (42.3)
Doctoral degree	6 (2.5)
**Age**	
< 22	23 (9.5)
22–26	173 (71.5)
27–31	33 (13.6)
> 32	13 (5.4)
**School Class^c^**	
P1	20.24 (6.72)
P2	18.1 (6.91)
P3	18.1 (6.37)
**Race^d^**	
Caucasian	213 (88.0)
African American	17 (7.0)
Hispanic	3 (1.2)
Asian	12 (5.0)
Native American	3 (1.2)

^a^ Two responders did not answer this question; ^b^ One responder did not answer this question; ^c^ p = 0.123 for comparison among classes; ^d^ Total is greater than 100% because four responders indicated multiple race.

**Table 2:** Perceived Stress Scale^12^ Results (*n* = 242).

	Never N (%)	Almost Never N (%)	Sometimes N (%)	Fairly Often N (%)	Very Often N (%)	Overall Response Score [Range 0–4] Mean (SD)
In the last month, how often have you been upset because of something that happened unexpectedly?	18 (7.4)	65 (26.8)	100 (41.3)	38 (15.7)	21 (8.7)	1.91 (1.03)
In the last month, how often have you felt that you were unable to control the important things in your life?	23 (9.5)	71 (29.3)	80 (33.1)	51 (21.1)	17 (7.0)	1.87 (1.07)
In the last month, how often have you felt nervous and “stressed”?	2 (0.8)	14 (5.8)	66 (27.8)	79 (32.6)	81 (33.4)	2.92 (0.95)
In the last month, how often have you felt confident about your ability to handle your personal problems?	1 (0.4)	8 (3.3)	77 (31.8)	99 (40.9)	57 (23.6)	1.61 (0.84)
In the last month, how often have you felt that things were going your way?	1 (0.4)	26 (10.7)	107 (44.2)	89 (36.8)	19 (7.9)	1.59 (0.80)
In the last month, how often have you found that you could not cope with all the things that you had to do?	32 (13.2)	84 (34.7)	68 (28.1)	45 (18.6)	13 (5.4)	1.68 (1.08)
In the last month, how often have you been able to control irritations in your life?	4 (1.7)	28 (11.6)	100 (41.3)	92 (38.0)	18 (7.4)	1.62 (0.85)
In the last month, how often have you felt that you were on top of things?	11 (4.5)	52 (21.5)	101 (41.7)	64 (26.4)	13 (5.4)	1.93 (0.94)
In the last month, how often have you been angered because of things that were outside of your control?	16 (6.6)	65 (26.9)	93 (38.4)	48 (19.8)	20 (8.3)	1.96 (1.03)
In the last month, have you felt difficulties were piling up so high that you could not overcome them?	29 (12.0)	70 (28.9)	75 (31.0)	43 (17.8)	25 (10.3)	1.86 (1.16)

**Table 3:** PSS-10 Score results ^a^.

	No. (%)	Overall Mean	SD
Male	72 (29.8%)	16.1	6.74
Female	170 (71.2)	19.6	6.29
Under 22 years of age	23 (9.5%)	21.4	6.54
22–31 years of age	206 (85.1%)	18.0	6.54
32 years of age and over	13 (5.4%)	21.9	6.71

^a:^ Possible range of scores: 0–40; p = 0.123 (for comparison among year in pharmacy school); PSS: Perceived Stress Scale; SD: Standard Deviation.

### 3.1. Stressors

Class assignments and completing electronic portfolios were the top stressors reported among our student pharmacists. There was a higher percentage of female participants that selected the items in the survey as stressors compared to males in all surveyed stress trigger items but one. Males had a higher percentage of stress than females concerning completing survey(s) from faculty or administration. A majority of students perceived IPPEs as a major stressor. Table 4 and Table 5 illustrate the stressors analyzed between genders and among age groups, respectively.

**Table 4:** Results ^a^ for stress triggers.

Survey Item	Male (*n* = 72)		Female (*n* = 170)		Total
	No. (%)	SD	No. (%)	SD	No. (%)
1. Class assignments	60 (83.3)	0.95	154 (90.6)	0.88	214 (88.4)
2. Completing electronic portfolios	48 (66.7)	1.2	143 (84.1)	1.01	191 (78.9)
3. Financial concerns	46 (63.9)	1.22	114 (67.1)	1.3	160 (66.1)
4. Introductory pharmacy practice experiences (IPPEs)	43 (59.7)	1.38	114 (67.1)	1.41	157 (64.9)
5. Family and relationships	35 (48.6)	1.12	111 (65.3)	1.12	146 (60.3)
6. Health or maintaining health	36 (50.0)	1.15	103 (60.6)	1.15	139 (57.4)
7. Academic competition	30 (41.7)	1.23	105 (61.8)	1.58	135 (55.8)
8. Extracurricular employment	37 (51.4)	1.26	92 (54.1)	1.27	129 (53.3)
9. Daily commute	25 (34.7)	1.29	81 (47.6)	1.32	106 (43.8)
10. Completing survey from faculty or administration	28 (38.9)	1.18	55 (32.4)	1.06	83 (34.3)
11. Completing capstone student surveys	12 (16.7)	0.88	49 (28.8)	1.05	61 (25.2)
12. Other	1 (1.4)	1.07	2 (1.2)	1	3 (1.2)

^a^ Results include surveys marked with response of the listed trigger very often, fairly often or sometimes causing stress.

**Table 5:** Results for stress triggers.

	< 22 [*N* = 23] (%)	22–31 [*N* = 206] (%)	32 & over [*N* = 13] (%)
1. Class assignments	21 (91.3)	179 (86.9)	13 (100)
2. Completing electronic portfolios	20 (86.9)	160 (77.7)	11 (84.6)
3. Financial concerns	15 (65.2)	134 (65.0)	11 (84.6)
4. Introductory pharmacy practice experiences (IPPEs)	18 (78.3)	129 (62.6)	10 (76.9)
5. Family and relationships	16 (69.6)	120 (58.3)	10 (76.9)
6. Health or maintaining health	13 (55.5)	115 (55.8)	11 (84.6)
7. Academic competition	16 (69.6)	111 (53.9)	8 (61.5)
8. Extracurricular employment	12 (52.2)	107 (51.9)	10 (76.9)
9. Daily commute	8 (34.8)	91 (44.2)	7 (53.8)
10. Completing survey from faculty or administration	7 (30.4)	71 (34.5)	5 (38.5)
11. Completing capstone student surveys	5 (21.7)	53 (25.7)	3 (23.1)
12. Other	0 (0)	3 (1.5)	0 (0)

### 3.2. Coping Mechanisms

Participants reported that spending time with family and friends as the best way to alleviate stress. Table 6 is an all-inclusive list of the surveyed coping mechanisms. Females had a higher percentage of participants that preferred shopping, sleeping/taking naps and/or eating as a means to improve stress levels. Playing video or online games, drinking alcohol and exercising were significantly higher in males as a means to relieve stress compared to females.

**Table 6:** Results^a^ for stress alleviators.

	Male (*n* = 72)	Female (n = 170)	Total (n = 242)
Stress Alleviator	No. (%)	No. (%)	No. (%)
1. Spending time with family and friends	62 (86.1)	162 (95.3)	224 (92.6)
2. Personal time alone	54 (75.0)	147 (86.5)	201 (83.1)
3. Eating	52 (72.2)	140 (82.4)	192 (79.3)
4. Exercising or playing sports	60 (83.3)	127 (74.7)	187 (77.3)
5. Sleeping or taking naps	46 (63.9)	131 (77.1)	177 (73.1)
6. Watching television	52 (72.2)	122 (71.8)	174 (71.9)
7. Shopping	14 (19.4)	105 (61.8)	119 (49.2)
8. Drinking alcohol	36 (50.0)	63 (37.1)	99 (40.9)
9. Study group	26 (36.1)	56 (32.9)	82 (33.9)
10. Reading at leisure	20 (27.8)	42 (24.7)	62 (25.6)
11. Playing video or online games	35 (48.6)	20 (11.8)	55 (22.7)
12. Nonprescription, herbal anti-anxiety or sleep aid meds	6 (8.3)	23 (13.5)	29 (12.0)
13. Prescription anti-anxiety medications or sleep aids	5 (6.9)	18 (10.6)	23 (9.5)
14. Other	6 (8.3)	17 (10.0)	23 (9.5)
15. Smoking cigarettes or use of other tobacco products	10 (13.9)	7 (4.1)	17 (7.0)

^a^ Results include surveys marked with response of the listed trigger very often, fairly often or sometimes causing stress.

## 4. Discussion

Our current study yielded a trend of females reporting higher stress, similar to the findings of Votta. Unlike the study by Votta, however, our study did not yield a negative correlation between year in the curriculum and stress score. Compared to Votta and Benau, it might be that our curriculum integration has led to less stress among students overall and equally rigorous years throughout the curriculum.

Marshall and colleagues reported mean PSS-14 scores for females and males were significantly different (28.1 *vs.* 22.4, respectively; *p* < 0.002) [2]. The authors also reported stressors and alleviators, with family and relationships ranking highest as a trigger and exercising ranking highest as an alleviator. Similarly, our current study found female students reporting higher levels of stress than their male counterparts. Strikingly different, however, we found the highest overall trigger in our current study was class assignments and the highest overall alleviator was spending time with family and friends. The timing of the survey in the Marshall study fell during the third week of a four-week pharmacotherapy block. It may be that differences in curriculum structure (blocks *versus* horizontal integration) resulted in differences in the degree to which class assignments precipitate stress among students. Our program horizontally integrates content within the same semester (*i.e.*, pharmaceutical sciences, pharmacotherapy, and integrated pharmacy laboratory course content are sequenced very intentionally to cover the same content around the same timeframe within a semester).

Gupchup and colleagues investigated the association between stress and health-related quality of life in first-, second-, and third-year student pharmacists [4]. Those authors also reported significantly higher stress scores for third-year students than for first-year students (148.60 *vs.* 133.43, respectively; *p* = 0.006). Our study findings were not consistent with Gupchup in this regard.

Dutta and colleagues studied stress in second-year student pharmacists in four different institutions [8]. The Derogatis Stress Profile (DSP) was used to measure stress in this study. The DSP associates scores above 63 to be experiencing psychiatric levels of distress. Scoring for the PSS-10 does not include excessive stress levels like the DSP. Therefore, our current study cannot determine those students who could experience stress at possible psychiatric levels.

Cohen and Janicki-Deverts reported psychological stress in a general population probability sample from 2009 using the PSS-10 (the same tool used in the current study) [13]. Men in this sample reported similar stress scores to those in our current study (15.52 *vs.* 15.9, respectively). However, women in Cohen’s study reported a lower stress score than the women in our study (16.14 *vs.* 19.6, respectively). Although age categories are divided into slightly different ranges between this previous study and our study (making exact comparisons difficult), there was lower stress reported by those less than 25 years of age in the Cohen study compared to our students in this study less than 22 years of age and our students 22 to 31 years of age (16.78 *vs.* 21.43 and 18.0, respectively). The difference in age categories between the two studies make comparisons difficult, yet the highest stress scores reported overall in the Cohen study was 17.46 by those ages 25–34 years. Our highest stress scores were for students ages 32 and averaged 21.92. Looking at this data overall, it does appear that student pharmacists report higher levels of perceived stress compared to the probability sample in the general population.

As a result of our findings, several initiatives have been taken at our institution. First, programming on student wellness and self-care delivered by the university’s social work faculty has been added to co-curricular programming during the first year of pharmacy school. Second, a focus group of nontraditional students was conducted to identify their unique needs. As a result of our study and the focus group results, a nontraditional student group has been formed and programming (networking, balancing multiple priorities) has been developed to help the nontraditional student and their families make the pharmacy school transition successfully. Third, because class assignments were identified as a key stressor, programming on time management as it relates to class assignments has been added to Orientation. Fourth, because finances were an important stressor, an information session on budgeting/financial planning (delivered by students for students) has been added to first year pharmacy student Orientation. Also related to finances, an “Applying for Financial Aid 101” booklet been developed for incoming students. Lastly, our methods for collecting and reviewing portfolio information are currently being re-evaluated by the school’s assessment personnel.

Limitations of this study include the fact that this study was performed at one private, church-based institution in the southeastern U. S. Therefore, the generalizability to other institutions may be limited and activities such as drinking alcohol may have been underreported. Additionally, our overall response rate was high at 68.4%; however, this ranges from 47.4% from the first-year class to 96.7% of the third-year class. The third-year response rate was due primarily to administration of the survey during an IPPE and, therefore, mandatory attendance. This IPPE activity was a one afternoon simulation activity on the university campus during what is otherwise a didactic semester. After explaining the study and the voluntary nature of participation, students’ anonymous responses were collected by non-faculty members. Additionally, the survey being administered in a large classroom environment could have led to students being able to view each other’s answers on the Scantron sheets used to collect the data. Another limitation is that the surveys were not administered at the same time. There were approximately 3–4 weeks between administration times for the three classes. In addition, when comparing the data to probability samples reported by Cohen and colleagues, different age categories were used [13]. This difference in age categories makes direct comparisons more challenging. Lastly, this study did not examine quality of life and, therefore, conclusions regarding stress and association with impact on quality of life cannot be made. Future study in that area may be warranted.

## 5. Conclusions

First-year female students reported the highest levels of stress than any other group in this study while second-year males reported the lowest levels of stress. Females in general perceived more stress than males, and the youngest and oldest age categories reported higher levels of stress. Given the results of our linear regression, additional variables not examined in this study (finances, health of students, *etc.*) may contribute to student stress and further study is warranted. Spending time with family and friends ranked highest as a stress alleviator and class assignments ranked highest as a stress trigger. Programming on stress reduction (particularly among female and nontraditional age students) appears warranted. Further study is needed to determine is such programming has an impact on stress levels after full implementation.
